# Sleep-related difficulties in healthy children and adolescents

**DOI:** 10.1186/s12887-021-02529-y

**Published:** 2021-02-16

**Authors:** Christiane Lewien, Jon Genuneit, Christof Meigen, Wieland Kiess, Tanja Poulain

**Affiliations:** 1 LIFE Leipzig Research Center for Civilization Diseases, Leipzig University, Philipp-Rosenthal-Strasse 27, 04103 Leipzig, Germany; 2 Department of Women and Child Health, Hospital for Children and Adolescents and Center for Pediatric Research (CPL), Leipzig University, Liebigstrasse 20a, 04103 Leipzig, Germany; 3 Pediatric Epidemiology, Department of Pediatrics, Medical Faculty, Leipzig University, Liebigstrasse 20a, 04103 Leipzig, Germany

**Keywords:** Sleep difficulties, Children’s Sleep Habits Questionnaire (CSHQ), Sleep Self Report (SSR), Age, Gender, Socioeconomic status (SES)

## Abstract

**Background:**

As sleep-related difficulties are a growing public health concern, it is important to gain an overview of the specific difficulty areas of the most vulnerable individuals: children. The current descriptive study presents the prevalence of sleep-related difficulties in two large samples of healthy children and adolescents and outlines the effects of age, gender, and socioeconomic status (SES) on various sleep-related difficulties.

**Methods:**

Participants were 855 4–9 year-old children (child sample) and 1,047 10–17 year-old adolescents (adolescent sample) participating 2011–2015 in the LIFE Child study, a population-based cohort study in Germany. Parents of the child participants completed the Children’s Sleep Habits Questionnaire (CSHQ), whereas adolescents self-administered the Sleep Self Report (SSR). Familial SES was determined by a composite score considering parental education, occupational status, and income. Multiple regression analyses were carried out to address the research question.

**Results:**

Among 4−9 year-old children, the mean bedtime was reported to be 8 p.m., the mean wake-up time 7 a.m., and sleep duration decreased by 14 min/year of age. 22.6 % of the children and 20.0 % of the adolescents showed problematic amounts of sleep-related difficulties. In the child sample, bedtime resistance, sleep onset delay, sleep-related anxiety, night waking, and parasomnia were more frequent in younger than older children. In the adolescent sample, difficulties at bedtime were more frequent among the younger adolescents, whereas daytime sleepiness was more prominent in the older than the younger adolescents. Considering gender differences, sleep-related difficulties were more frequent among boys in the child sample and among girls in the adolescent sample. Lower SES was associated with increased sleep-related difficulties in the adolescent, but not the child sample.

**Conclusions:**

The present results report sleep-related difficulties throughout both childhood and adolescence. Gender differences can already be observed in early childhood, while effects of SES emerge only later in adolescence. The awareness for this circumstance is of great importance for pediatric clinicians who ought to early identify sleep-related difficulties in particularly vulnerable individuals.

## Background

Healthy sleep habits in children are crucial as sleep promotes healthy physical and mental child development. Moreover, sleep disorders have been linked to a variety of cognitive, behavioral, emotional, and physical health problems [1–4]. It is therefore alarming that sleep duration has decreased, while the frequency of sleep problems has increased in recent decades [1, 5–9]. Prevalence rates of sleep problems range from 15 to 44 % [10]; in Germany and the United States, 20–25 % have been previously reported [11, 12].

It is well established that children’s individual sleep habits change during childhood and adolescence. With increasing age, bedtimes become later and sleep durations reduce [7, 13]. Furthermore, specific sleep-related difficulty areas can be attributed to specific age groups. The younger the child, the more bedtime resistance and difficulties sleeping through the night are reported; the older the child, the more they report difficulties falling asleep and daytime sleepiness [11, 13, 14].

Uncertainty still exists regarding the relationship between gender and sleep-related difficulties. Data from several studies suggest that, in adolescence, girls tend to report sleep difficulties more frequently than boys [1, 6, 7, 11]. One possible explanation is suspected to lie in the onset of puberty and consequent gender differences [15, 16]. In contrast to adolescents, the results in children are inconsistent. Whereas some studies observed more sleep difficulties among boys than girls [17], others found no gender difference [11], and yet others reported more frequent difficulties among girls than boys [13].

A further influential factor on child sleep discussed in the literature is the familial socioeconomic status (SES). Singh and Kenney [5] found up to 43 % higher adjusted odds of serious sleep problems for children and adolescents from neighborhoods with the most unfavorable social conditions compared to their counterparts from the most favorable neighborhoods. Whereas associations between lower social status and poorer sleep outcomes have been shown in several samples of older children and adolescents [18–20], previous findings on younger children are inconsistent. Whereas some studies were not able to identify any associations between SES and children’s sleep [17, 21], others reported more inconsistent bedtime routines and sleep behavior problems in socially disadvantaged children [22, 23].

The aims of the present study were to characterize sleep habits (bedtime, wake-up time, sleep duration) in children, to determine the prevalence of problematic amounts of sleep-related difficulties, and to analyze the effects of age, gender, and SES on sleep habits and specific sleep-related difficulties in children and adolescents. Since most previous studies have explored small samples of children in specific age groups, this study aimed to extend the literature by presenting data from two large samples of healthy German children and adolescents. In contrast to previous studies mainly focusing on difficulties falling asleep and sleeping through the night, the present study investigated several different sleep domains: sleep-related anxiety, sleep-disordered breathing, sleep onset delay, night waking, daytime sleepiness, sleep duration, parasomnia, and bedtime resistance. Despite the extensive research carried out on the effects of age, gender, and SES on sleep-related difficulties, the results remain unclear, especially regarding childhood. We expected to confirm age-related differences throughout childhood and gender effects in adolescents. Furthermore, we hypothesized the SES to impact sleep-related difficulties in children as well as adolescents.

## Methods

### Participants

Data for the present project were collected within the LIFE Child study, an ongoing cohort study conducted in Leipzig, Germany. The aim of LIFE Child is to investigate healthy child development and the etiology of civilization diseases [24, 25]. All children and adolescents without chronic, chromosomal, or syndromal diseases are eligible to participate. They are recruited via advertising at public health centers, hospitals, and schools in the surroundings of Leipzig. LIFE Child was prepared in compliance with the Helsinki Declaration and with the agreement of the Ethics Committee of the Medical Faculty of the Leipzig University (Reg. No. 264-10-19,042,010). Written informed consent is obtained from all parents and from children from the age of 12.

The assessment of sleep and related parameters represents only one part of the large LIFE Child study, with data collected mainly between 2011 and 2015. In this time period, 1,902 children and adolescents provided complete information on sleep habits and sleep-related difficulties and could therefore be included in the present analyses. In cases where records from several years were available for the same participant, one visit was selected at random to prevent bias caused by multiple visits. For the present analyses, two samples were distinguished by age. The child sample comprised 855 4−9 year-olds and the adolescent sample comprised 1,047 10−17 year-olds.

### Measures

#### Children’s Sleep Habits Questionnaire (CSHQ)

In the child sample, the Children’s Sleep Habits Questionnaire (CSHQ) was used to acquire parent-reported information on children’s sleep-related difficulties (sleep-related anxiety, sleep-disordered breathing, sleep onset delay, night waking, daytime sleepiness, sleep duration, parasomnia, and bedtime resistance) [26, 27]. For each question, parents are asked to assess on a three-point scale if the described behavior occurs “rarely” (0–1 times), “sometimes” (2–4 times), or “usually” (5–7 times) during a typical week. For each sleep-related difficulty subscale, the child receives a score indicating the extent of difficulties. All subscale scores can be combined to a total difficulties score. This score ranges from 33 to 99, with higher scores indicating more sleep-related difficulties. In a representative German sample, a total score of 47 reflected the 90th percentile [27]. We therefore considered values above to reflect problematic amounts of sleep-related difficulties. In the present study, cronbach’s α coefficients ranged between 0.47 and 0.74 for the CSHQ subscales. These internal consistencies are comparable to the original study [27].

In the CSHQ, parents are also asked to state the habitual bedtime, the morning wake-up time and the usual sleep duration (day and night) of their child. However, due to item-specific missing data on sleep habits, these analyses were performed with fewer observations (bedtime analysis *n* = 675, wake-up time analysis *n* = 735, sleep duration analysis *n* = 619).

#### Sleep Self Report (SSR)

In the adolescent sample, the Sleep Self Report (SSR) was self-administered [28, 29]. This questionnaire also aims to identify potential sleep-related difficulties with a focus on difficulties at bedtime, sleep behavior difficulties, and daytime sleepiness. The validation for the SSR was originally performed for 7–12 year-olds; however, it is also widely applied until the age of 18 [4, 30]. The response options are designed in the same three-point scale as the CSHQ and the respondents’ scores can be combined to a total difficulties score ranging from 18 to 54. A total score of 31 corresponded to the 90th percentile in a representative German sample [28]. We therefore considered higher values to reflect problematic amounts of sleep-related difficulties. In the present sample, cronbach’s α coefficients ranged between 0.50 and 0.62 for the SSR subscales, which is comparable to the original study [28].

#### Socioeconomic status (SES)

Information on socioeconomic status (SES) was collected via a parental questionnaire adapted to the original version used in the German Health Interview and Examination Survey for Children and Adolescents (KiGGS), a national survey on child health in Germany [31]. For the assessment of a family’s socioeconomic status, family net income, parental education, and occupational status were accordingly subsumed under a composite score which can range from 3 to 21 [32]. This score allows a classification of families into low (3–8.4), middle (8.5–15.4), and high (15.5–21) SES [31, 32]. According to a large representative German cohort study, a representative study sample should include 20 % of participants from low SES, 60 % from middle SES, and 20 % from high SES [31].

### Analyses

Statistical analysis and visualization were performed using R version 3.6.1 [33]. To explore sleep habits in the child sample, multiple linear regression models were performed. In a first model, either bedtime or wake-up time was included as the dependent variable and age (as continuous measure), gender (as two-level categorical measure), and SES (as continuous measure) were included as independent variables. In a following model, the sleep duration was analyzed depending on bedtime, age, gender, and SES.

The prevalence of problematic amounts of sleep-related difficulties was indicated by total scores > 47 and > 31 in the child sample and in the adolescent sample, respectively. A logistic regression analysis was performed with each sample to investigate the prevalence odds (as the dependent variable) depending on the three independent variables age, gender, and SES.

Furthermore, in both the child and the adolescent sample, multiple linear regression models were performed to investigate associations of the scores on the different sleep-related difficulty subscales (as dependent variables) with age, gender, and SES (as independent variables).

For all regression models, the significance level *α* was determined to be 5 % and strengths of associations were described by non-standardized regression coefficients *b* or odds ratios (*OR*). In moderator analyses, all models were checked for interactions between the independent variables and additionally tested for their model quality (good model quality was reflected by variance inflation factors < 5). Herewith, no moderator effects were observed.

## Results

### Descriptive statistics

Participant characteristics are summarized in Table 1. In the child sample, the mean SES was 13.4 ± 3.39 and showed a clear tendency towards higher social strata. In the adolescent sample, this tendency was less pronounced (mean SES = 12.7 ± 3.47). Whereas middle and high SES families were well represented in the present samples, low SES families were underrepresented with 9.6 % in the child sample and 14.0 % in the adolescent sample (in comparison with a representative German cohort sample [31]).

**Table 1:** Participant characteristics of the child sample and the adolescent sample

	Child sample (*n* = 855)		Adolescent sample (*n* = 1,047)	
	*n (%)*	*mean (SD)*	*n (%)*	*mean (SD)*
Gender				
Male	434 (50.8)		508 (48.5)	
Female	421 (49.2)		539 (51.5)	
Age (y)		7.2 (1.79)		13.2 (2.16)
4–5	249 (29.1)			
6–7	251 (29.4)			
8–9	355 (41.5)			
10–11			365 (34.9)	
12–13			288 (27.5)	
14–15			260 (24.8)	
16–17			134 (12.8)	
SES ^a^		13.4 (3.39)		12.7 (3.47)
Low	82 (9.6)		147 (14.0)	
Middle	499 (58.4)		640 (61.1)	
High	274 (32.0)		260 (24.8)	
	*n (%) *^*b*^	*missing (%)*	*n (%) *^*b*^	*missing (%)*
School education		232 (27.1)		455 (43.4)
Low	17 (2.7)		30 (5.1)	
Middle	179 (28.7)		224 (37.9)	
High	413 (66.3)		318 (53.8)	
Living with child		42 (4.91)		270 (25.8)
Mother	803 (98.8)		755 (97.2)	
Father	601 (73.9)		522 (67.2)	
Own nursery	684 (84.1)	42 (4.91)	716 (92.3)	271 (25.9)

^a ^Low SES: SES 3–8.4; Middle SES: SES 8.5–15.4; High SES: SES 15.5–21

^b ^Percentages were calculated excluding missing data

In both the child sample (66.3 %) and the adolescent sample (53.8 %), most parents (either the mother or the father) had attained the highest German school degree (‘Abitur’). Both children and adolescents lived with their biological mothers in nearly all cases, while the biological father lived in the child’s home less often (in 73.9 % of the cases in the child sample and in 67.2 % in the adolescent sample). In the child sample, 84.1 % of children had their own nursery. Among the adolescents, this was the case in 92.3 %.

### Sleep duration in the child sample

**Fig. 1:**
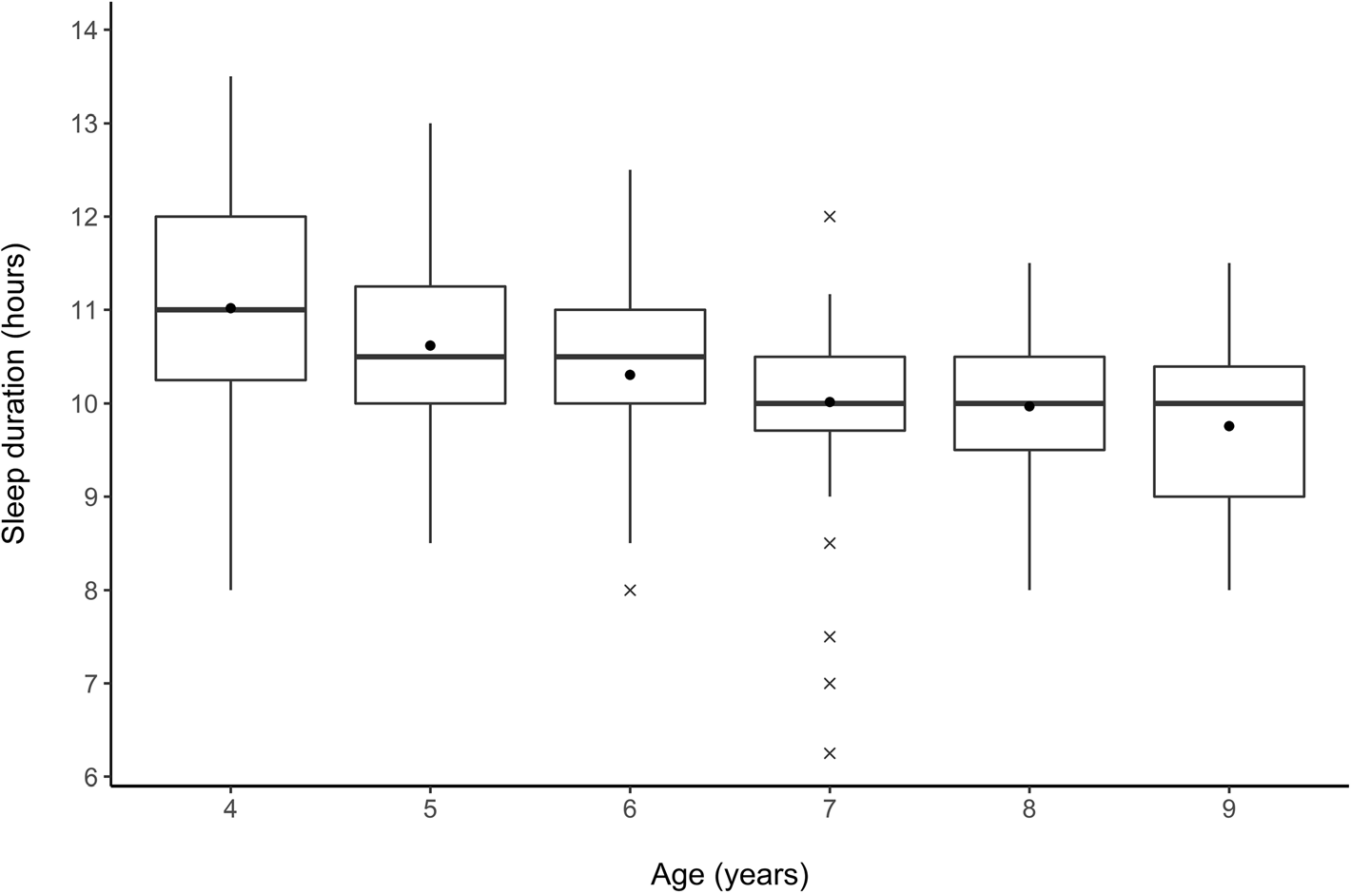
Sleep duration by age in the child sample. *Note.* Sleep duration by age according to the Children’s Sleep Habits Questionnaire (CSHQ), *n* = 855. The box indicates the first and third quartile and the median, and the point indicates the arithmetic mean. The whiskers represent the range between minimum and maximum, outliers are represented by a cross

In the child sample, the mean bedtime reported by parents was 7:54 p.m. (SD = 29 minutes) and the mean wake-up time 6:59 a.m. (SD = 46 minutes). Analyses with either bedtime or wake-up time and age, gender, and SES resulted in some significant associations, however, with very small effect sizes (age − bedtime: *b* = 0.04 [95 % CI: 0.02; 0.06], *p* < 0.001; age − wake-up time: *b* = -0.04 [95 % CI: -0.07; -0.008], *p* = 0.014; SES − bedtime: *b* = 0.02 [95 % CI: 0.01; 0.03], *p* = 0.002). The mean sleep duration was reported as 10.2 hours (SD = 59 minutes). Shorter sleep duration was associated with later bedtime (*b* = -0.50 [95 % CI: -0.66; -0.33], *p* < 0.001). Furthermore, the sleep duration decreased by 14 minutes per year of age (*b* = -0.22 [95 % CI: -0.26; -0.18], *p* < 0.001, see Fig. 1). Analyses with gender and SES did not reach statistical significance.

### Problematic amounts of sleep‐related difficulties in the child and the adolescent sample

**Fig. 2:**
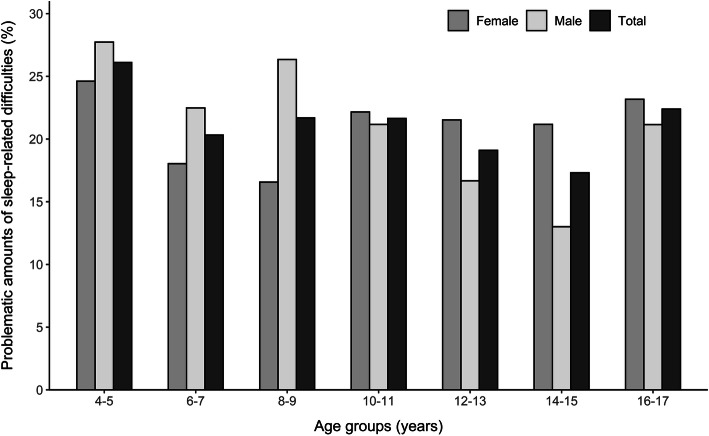
Prevalence of problematic amounts of sleep-related difficulties by age and gender. *Note.* Prevalence of problematic amounts of sleep-related difficulties by age and gender in the child sample (age groups 4−5, 6−7, and 8−9) and in the adolescent sample (age groups 10−11, 12−13, 14−15, and 16−17)

The mean total difficulties scores in the child and adolescent sample, according to the CSHQ and the SSR, were 42.5 (SD = 5.66, range 33–99) and 26.8 (SD = 4.62, range 18–54), respectively. In the child sample, 22.6 % of participants reported problematic amounts of sleep-related difficulties. Among adolescents, the prevalence was 20.0 %. For the distribution of the prevalence rates in detail, see Fig. 2. In logistic regression analyses with the three independent variables age, gender, and SES, significant age effects were found neither for the children nor the adolescents. In contrast, gender differences were observed. In the child sample, the prevalence of problematic amounts of sleep-related difficulties was significantly higher in boys than in girls (*OR* = 0.70 [95 % CI: 0.50; 0.96], *p* = 0.029). In the adolescent sample, in contrast, the risk was higher in girls than in boys, albeit only marginally statistically significant (*OR* = 1.30 [95 % CI: 0.95; 1.76], *p* = 0.098). In the adolescents, a high SES was associated with a lower prevalence of problematic amounts of sleep-related difficulties (*OR* = 0.95 [95 % CI: 0.91; 0.99], *p* = 0.020). In the children, no significant association was found.

### Associations between sleep‐related difficulties and age, gender, and SES

#### Child sample

**Fig. 3:**
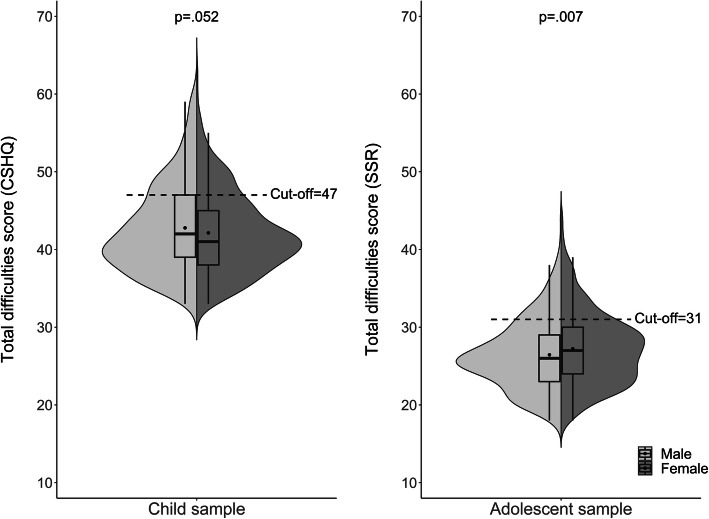
Distribution of the total difficulties score by gender in the child sample and the adolescent sample. *Note.* Distribution of the total difficulties score by gender in the child sample (on the left, according to the Children’s Sleep Habits Questionnaire [CSHQ], score range = 33−99, *n* = 855) and the adolescent sample (on the right, according to the Sleep Self Report [SSR], score range = 18−54, *n* = 1,047). Shaded areas show split violin plots of the density function, with inserted boxes indicating the first and third quartile and the median, and with the point indicating the arithmetic mean. The displayed *p*-values are derived from linear regression analysis. Scores above the cut-off value indicate problematic amounts of sleep-related difficulties

Associations identified between the scores on the different sleep-related difficulty scales of the CSHQ and age, gender, and SES are summarized in Table 2. Significant associations with child age showed that bedtime resistance (*b* = -0.08 [95 % CI: -0.14; -0.01], *p* = 0.020), sleep onset delay (*b* = -0.09 [95 % CI: -0.11; -0.06], *p* < 0.001), sleep-related anxiety (*b* = -0.11 [95 % CI: -0.16; -0.06], *p* < 0.001), night waking (*b* = -0.11 [95 % CI: -0.15; -0.06], *p* < 0.001), and parasomnia (*b* = -0.07 [95 % CI: -0.12; -0.01], *p* = 0.013) were more frequent in younger children. Older children, in contrast, were reported to experience more daytime sleepiness than younger children (*b* = 0.19 [95 % CI: 0.09; 0.30], *p* < 0.001). However, no significant association between the total difficulties score and age was observed. Considering gender differences, boys scored significantly higher than girls on the subscales of parasomnia (*b* = -0.23 [95 % CI: -0.42; -0.04], *p* = 0.020) and night waking (*b* = -0.17 [95 % CI: -0.32; -0.01], *p* = 0.036). For the total difficulties score, this gender difference was marginally significant (*b* = -0.75 [95 % CI: -1.51; 0.01], *p* = 0.052). Together with the aforementioned gender differences in the prevalence of problematic amounts of sleep-related difficulties, these results imply that in childhood, boys suffer from more sleep-related difficulties than girls (see also Fig. 3). With regard to the relationship between SES and sleep-related difficulties, negative associations were found for the subscales of sleep-related anxiety (*b* = -0.04 [95 % CI: -0.07; -0.02], *p* = 0.002), sleep-disordered breathing (*b* = -0.02 [95 % CI: -0.04; -0.01], *p* = 0.005), and parasomnia (*b* = -0.06 [95 % CI: -0.09; -0.04], *p* < 0.001). These associations indicate that children growing up in families with higher SES experience fewer difficulties in these areas. The association between SES and the total difficulties score showed the same trend, however, statistical significance was not reached.

**Table 2:** Mutually adjusted associations of sleep-related difficulties with age, gender, and socioeconomic status (SES) in the child sample

	Dependent variables					
	Sleep-related anxiety		Sleep-disordered breathing		Sleep onset delay	
Independent variables	*b* (95% CI)	*p*	*b* (95% CI)	*p*	*b* (95% CI)	*p*
Age	-0.11 (-0.16; -0.06)	< .001	-0.02 (-0.05; 0.01)	.167	-0.09 (-0.11; -0.06)	< .001
Gender ^a^	-0.18 (-0.36; 0.002)	.053	-0.05 (-0.15; 0.05)	.354	0.01 (-0.09; 0.11)	.840
SES	-0.04 (-0.07; -0.02)	.002	-0.02 (-0.04; -0.01)	.005	-0.001 (-0.02; 0.01)	.920
	Night waking		Daytime sleepiness		Sleep duration	
	*b* (95% CI)	*p*	*b* (95% CI)	*p*	*b* (95% CI)	*p*
Age	-0.11 (-0.15; -0.06)	< .001	0.19 (0.09; 0.30)	< .001	0.03 (-0.02; 0.08)	.231
Gender ^a^	-0.17 (-0.32; -0.01)	.037	-0.07 (-0.44; 0.31)	.726	-0.10 (-0.27; 0.08)	.287
SES	0.01 (-0.01; 0.04)	.218	0.05 (-0.01; 0.10)	.085	-0.003 (-0.03; 0.02)	.809
	Parasomnia		Bedtime resistance		Total difficulties score	
	*b* (95% CI)	*p*	*b* (95% CI)	*p*	*b* (95% CI)	*p*
Age	-0.07 (-0.12; -0.01)	.013	-0.08 (-0.14; -0.01)	.020	-0.18 (-0.39; 0.03)	.096
Gender ^a^	-0.23 (-0.42; -0.04)	.020	-0.03 (-0.27; 0.20)	.788	-0.75 (-1.51; 0.01)	.052
SES	-0.06 (-0.09; -0.04)	< .001	-0.03 (-0.07; 0.0002)	.052	-0.10 (-0.21; 0.02)	.092

*Note.* According to the Children’s Sleep Habits Questionnaire, *n* = 855

^a ^Reference = female gender

#### Adolescent sample

Associations found between the scores on the different sleep-related difficulty scales of the SSR and age, gender, and SES are summarized in Table 3. The analyses revealed that older adolescents reported more daytime sleepiness (*b* = 0.13 [95 % CI: 0.09; 0.18], *p* < 0.001) and fewer difficulties at bedtime than younger adolescents (*b* = -0.08 [95 % CI: -0.16; -0.01], *p* = 0.030). However, no significant association between the total difficulties score and age was observed. Compared to boys, girls scored significantly higher on the sleep behavior difficulty scale (*b* = 0.46 [95 % CI: 0.24; 0.68], *p* < 0.001) and on the total difficulties score (*b* = 0.78 [95 % CI: 0.22; 1.33], *p* = 0.007). This result indicates, along with the aforementioned gender differences in the prevalence of problematic amounts of sleep-related difficulties, that adolescent girls suffer from more sleep-related difficulties than boys (see also Fig. 3). Regarding the associations between SES and sleep-related difficulties, a lower SES was associated with a higher total difficulties score (*b* = -0.12 [95 % CI: -0.20; -0.04], *p* = 0.004), more bedtime difficulties (*b* = -0.07 [95 % CI: -0.11; -0.02], *p* = 0.004), and more sleep behavior difficulties (*b* = -0.04 [95 % CI: -0.07; -0.005], *p* = 0.025). These associations indicate that adolescents growing up in families with a higher SES experience fewer sleep-related difficulties.

**Table 3:** Mutually adjusted associations of sleep-related difficulties with age, gender, and socioeconomic status (SES) in the adolescent sample

	Dependent variables			
	Bedtime difficulties		Sleep behavior difficulties	
Independent variables	*b* (95% CI)	*p*	*b* (95% CI)	*p*
Age	-0.08 (-0.16; -0.01)	.030	-0.03 (-0.09; 0.02)	.182
Gender ^a^	0.22 (-0.10; 0.54)	.180	0.46 (0.24; 0.68)	< .001
SES	-0.07 (-0.11; -0.02)	.004	-0.04 (-0.07; -0.005)	.025
	Daytime sleepiness		Total difficulties score	
	*b* (95% CI)	*p*	*b* (95% CI)	*p*
Age	0.13 (0.09; 0.18)	< .001	0.02 (-0.11; 0.15)	.798
Gender ^a^	0.10 (-0.08; 0.28)	.282	0.78 (0.22; 1.33)	.007
SES	-0.01 (-0.04; 0.01)	.280	-0.12 (-0.20; -0.04)	.004

*Note.* According to the Sleep Self Report, *n* = 1,047

## Discussion

This descriptive study recruited from the general population investigated sleep habits and sleep-related difficulties in two large samples of healthy children and adolescents. Furthermore, associations with age, gender, and SES were explored. It is one of only a few publications to present data from early childhood to late adolescence from one representative cohort study.

### Sleep duration in children

The mean bedtime and wake-up time in children were reported to be 7:54 p.m. and 6:59 a.m., respectively, which is comparable to results from a previous Dutch study that resembles the present setting in the socio-cultural background of participants and the implementation of the study [13]. Similar to other studies, gender differences were identified for neither the bedtime nor wake-up time [9, 13, 34]. With increasing child age, these studies report later bedtimes with similar wake-up times. In our sample, the differences in bedtime or wake-up time between children of different age were negligibly small. Similar wake-up times might be the reflection of regular school and parental work schedules. One reason bedtimes were only slightly postponed in our sample could lie in the acquisition method; the CSHQ asks the parents about the bedtime, yet this could vary from the actual sleep onset. Older children might go to bed at the same time as younger children but might take longer to fall asleep.

The mean sleep duration of 4–9 year-olds was reported as 10.2 hours and thereby meets the recommendations of the National Sleep Foundation which advises 10–13 hours of sleep for preschoolers and 9–11 hours for school-aged children [35]. Consistent with previous findings [9, 36], our results suggest that sleep duration declines as children grow older. This decrease appears to be multifactorial, influenced by biological processes, the effects of school and leisure activities competing for sleep, media use, and pubertal changes [36]. In accordance with the results from another large German study of 17,641 children [11], sleep duration did not differ depending on child gender.

In the present study, wake-up time and sleep duration were unaffected by SES, as was bedtime, given that the effect size was negligibly small. Reports in the literature of socioeconomic effects are inconsistent. Previous research in preschool-aged children suggests that socioeconomic variables do not account for differences in sleep duration [37]. Yet, several studies suggest poorer sleep outcomes for children with a lower social background in that bedtimes would be later, sleep durations shorter, and bedtime routines less consistent than in children from more advantaged households [19, 22, 23]. Beyond our analyses on SES effects, cross-cultural differences might mediate the association between social status and sleep or might stand alone as an influencing factor and should therefore be mentioned. Differences in genetic background, lifestyle (e.g., in health behavior, use of leisure time), environmental factors (e.g., in number of household members, room-sharing), cultural practices (e.g., in parenting behavior, parent co-sleeping, napping) as well as in cognitions and attitudes towards normal versus impaired sleep, may also account for differences in the research [13, 34, 37, 38]. Further studies will need to be conducted to clarify the association of sleep habits and a family’s SES (more on the effects of SES in the following section).

### Sleep‐related difficulties in children and adolescents

Overall, problematic amounts of sleep-related difficulties were frequently reported by participants across all age groups with 22.6 % of children and 20.0 % of adolescents affected. A multitude of prevalence rates are described in the literature, ranging from 15 to 44 % [10]. It must be noted that prevalence rates change due to cultural differences, heterogeneous definitions of impaired sleep, different methodology, and the inclusion of different age groups. Compared to the German validation studies of the CSHQ and SSR in which by definition of the cut-off value 10 % of children received problematic scores [27, 28], the prevalence rates showed a twofold increase. As the aforementioned studies were conducted 10 years ago, this discrepancy might reflect the increase in sleep difficulties indicated by previous research [1, 5, 6].

For both children and adolescents, only specific sleep-related difficulty areas were associated with child age. In children, more resistance at bedtime, sleep-related anxiety, night waking, and parasomnia were reported; in adolescents, more daytime sleepiness. These results corroborate the findings of previous work and might reflect the normal development of a child [11, 13, 14]. However, in contrast to previous studies reporting more difficulties falling asleep in older versus younger children and adolescents [11, 13], we observed less sleep onset delay in the older versus the younger children as well as fewer difficulties at bedtime in the older versus the younger adolescents. A reason could be that bedtimes are mainly parent-regulated in younger children whereas older children go to bed independently. Previous research was able to show that as well as parental regulation of bedtimes, difficulties falling asleep also reduced with increasing age [39].

The present results revealed a gender difference in sleep-related difficulties: In children, these were more frequent among boys, while in adolescents among girls. In childhood and pre-puberty, previous results on gender differences have been inconsistent [11, 13, 17]. Since parental reports were used for children, one possible explanation for the present results might lie in a parental tendency to rate sleep and behavioral difficulties worse in boys than girls [4, 40]. Previous studies have suggested that boys are more likely to exhibit externalizing behavior, while girls are more likely to exhibit internalizing behavior [40, 41]. When the children cannot sleep at night, boys might be more likely to receive attention through acting-out behavior, while girls might be more compliant and, therefore, attract less attention [41]. As far as adolescents are concerned, there is consensus in the current literature that sleep problems occur more frequently in girls [1, 6, 11]. Several studies have considered the cause in pubertal differences between girls and boys of the same age [15, 16]. Hormonal differences, however, might not suffice as an explanation since comprehensive research using polysomnography in 0–18 year-olds found that sleep parameters differed greatly between subjects of different Tanner stages, yet did not observe any gender difference [42]. Please note that the analysis of different Tanner stages was beyond the scope of the present paper.

In this study, a low SES in adolescents was associated with increased sleep-related difficulties. In children, solely the areas of sleep-related anxiety, sleep-disordered breathing, and parasomnia were inversely associated with SES. Notably, symptoms of parasomnia (e.g., sleepwalking, talking in sleep, bruxism, nightmares) and sleep-disordered breathing in children have previously been associated with low familial SES [43, 44]. However, with regard to the other sleep parameters and sleep-related difficulties, previous findings in children have been inconsistent [17, 21, 22]. In line with our findings in adolescents, a wide range of previous studies demonstrated the association of a low familial SES with increased sleep difficulties in adolescence [5, 18, 20]. As possible mediators of this association, the existing literature suggests greater pre-sleep worries, disruptive sleep conditions, poorer neighborhood conditions, academic demands, and a less healthy lifestyle (e.g., increased media usage, physical inactivity) in lower SES families [5, 18, 19, 45]. A reason the association between SES and sleep-related difficulties was observed in adolescents but not in children, might be that these possible mediators become more relevant as children grow older [5, 45–47]. A further reason might lie in the methodology (parent report versus self-report). Parent reports rely highly on parental awareness and attitudes concerning their child’s sleep and health issues [48, 49] which might also differ between families from different socioeconomic strata.

### Limitations

The present study was able to investigate a variety of sleep-related difficulties and is noteworthy due to its large sample size, broad age range, and the inclusion of parental as well as self-reports. Nonetheless, some limitations should be considered. Given that the study mainly included children from a single large city in eastern Germany and that children from families with a lower social status were underrepresented, conclusions regarding the overall population of children should be made with caution. Since the latest data were acquired in 2015, the results are not entirely of recent date. Further limitations concern the acquisition method. In order to obtain the most reliable data according to age, parent reports for children and self-reports for adolescents were used. Therefore, analyses had to be carried out separately and interpretations spanning the whole range from early childhood to adolescence should be made with caution. An additional limitation is that questions on sleep habits were not asked separately for weekdays and weekend. Sleep duration as well as other sleep habits might differ significantly between weekdays and weekend.

Further research is required to clarify the effects of gender and SES on sleep-related difficulties. In particular, differences between childhood and adolescence should be investigated in more detail.

## Conclusions

Overall, the present results report sleep-related difficulties throughout both childhood and adolescence. Furthermore, gender differences can already be observed in early childhood, while effects of SES emerge only later in adolescence. Our findings suggest that young boys, adolescent girls, and adolescents from lower social strata are at special risk to experience sleep-related difficulties. The awareness of this circumstance is of great importance for pediatric clinicians who ought to early identify sleep-related difficulties in particularly vulnerable individuals.

## Data Availability

The data sets on which the present study is based are not publicly accessible since the publication of data is not covered by the informed consent of study participants. Potentially sensitive information is collected in the context of the LIFE Child study; therefore, the data protection concept requires that all researchers interested in accessing data sign a project agreement. Researchers interested in gaining access to the data collected in the LIFE Child study may contact the committee on data use and access (dm@life.uni-leipzig.de).

## Abbreviations

CSHQ: Children’s Sleep Habits Questionnaire
SSR: Sleep Self Report
SES: Socioeconomic status
